# Hunting in Bioluminescent Light: Vision in the Nocturnal Box Jellyfish *Copula sivickisi*

**DOI:** 10.3389/fphys.2016.00099

**Published:** 2016-03-30

**Authors:** Anders Garm, Jan Bielecki, Ronald Petie, Dan-Eric Nilsson

**Affiliations:** ^1^Marine Biological Section, Department of Biology, University of CopenhagenCopenhagen, Denmark; ^2^Department of Ecology evolution and Marin Biology, University of California, Santa BarbaraSanta Barbara, CA, USA; ^3^Vision Group, Department of Biology, Lund UniversityLund, Sweden

**Keywords:** cubozoa, night active, eyes, spectral sensitivity, foraging

## Abstract

Cubomedusae all have a similar set of six eyes on each of their four rhopalia. Still, there is a great variation in activity patterns with some species being strictly day active while others are strictly night active. Here we have examined the visual ecology of the medusa of the night active *Copula sivickisi* from Okinawa using optics, morphology, electrophysiology, and behavioral experiments. We found the lenses of both the upper and the lower lens eyes to be image forming but under-focused, resulting in low spatial resolution in the order of 10–15°. The photoreceptor physiology is similar in the two lens eyes and they have a single opsin peaking around 460 nm and low temporal resolution with a flicker fusion frequency (fff) of 2.5 Hz indicating adaptions to vision in low light intensities. Further, the outer segments have fluid filled swellings, which may concentrate the light in the photoreceptor membrane by total internal reflections, and thus enhance the signal to noise ratio in the eyes. Finally our behavioral experiments confirmed that the animals use vision when hunting. When they are active at night they seek out high prey-concentration by visual attraction to areas with abundant bioluminescent flashes triggered by their prey.

## Introduction

Within Cnidaria a small group, the cubozoans, have diverged to evolve an elaborate visual apparatus along with an according expansion of their nervous systems (Satterlie, 1979; Garm et al., 2007). The cubomedusae, or box jellyfish, all possess a similar set of 24 eyes distributed on four sensory structures called rhopalia. Each rhopalium holds eyes of four morphologically distinct types, the upper lens eye, the lower lens eye, the pit eyes, and the slit eyes, and offer a clear example of special purpose eyes (Yamasu and Yoshida, 1976; Pearse and Pearse, 1978; Matsumoto, 1995; Nilsson et al., 2005; Garm et al., 2008). The optics have been investigated only in two species, *Tripedalia cystophora* and *Chiropsella bronzie*. In these species, the lens eyes provide low spatial resolution in the order of 10° or worse (Nilsson et al., 2005; O'Connor et al., 2009). This does not allow visually guided hunting for prey. Instead they use vision to seek out habitats with high prey densities. This is best understood for the Caribbean species, *T. cystophora*, which feed on copepods accumulating in light shafts between the mangrove prop roots (Buskey, 2003; Garm et al., 2011). They avoid the dark roots but are attracted to the light shafts where the positively phototactic copepods gather. Once in the right habitat they hunt passively with extended and trailing tentacles and the actual prey capture is no different from the typical scypho- and hydromedusa which rely on the prey accidentally contacting a tentacle.

**Figure 1:**
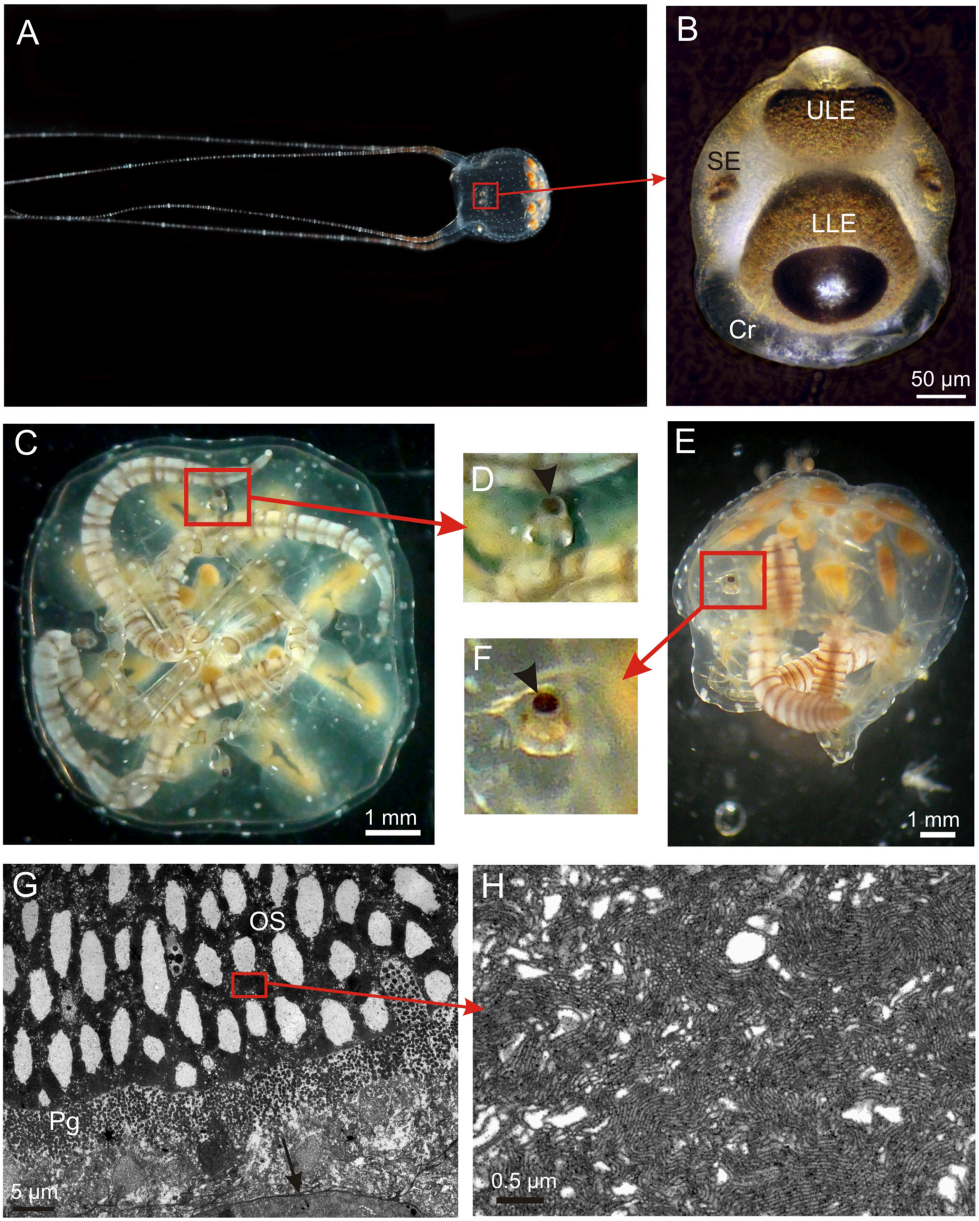
**The visual system of ***Copula sivickisi***. (A)** An adult male fishing with outstretched tentacles. Framed area indicates location of one of the rhopalia. **(B)** Close up of a rhopalium showing four of the six eyes. Note the large crystal (Cr) distally. **(C)** Resting medusae lying upside down but the rhopalia still orient with the upper lens eye pointing straight upwards due to the heavy crystal. **(D)** Close up of framed area in **(C)** showing upright rhopalium, arrowhead indicates upper lens eye. **(E)** Medusa laying on the side still having upright rhopalia. **(F)** Close up of framed area in **(E)** showing upright rhopalium, arrowhead indicates upper lens eye. **(G)** TEM micrograph of photoreceptors in the lower lens eye. The outer segments (OS) are modified cilia, and the photoreceptors also contain screening pigment (Pg). Note apparent holes in the outer segments. **(H)** Close up of OS showing the cilia projecting densely packed microvilli. LLE, lower lens eye; SE, slit eye; ULE, upper lens eye.

The small eyes (pupil diameter <100 μm) of *T. cystophora* agree with their diurnal activity pattern: they are only found hunting between the prop roots during the day and rest on the bottom at night (Garm et al., 2012). Still, in all other examined species it seems to be different. Several species are found actively swimming both day and night, but unfortunately most of the data originates from tank experiments which might induce artificial behaviors (Yatsu, 1917; Satterlie, 1979). In one case, the Australian *Chironex fleckeri*, individuals have been tagged in the wild, and the tracking revealed that this species has great variations between individuals but at least some were just as active during dark hours as during the day (Gordon and Seymour, 2009). Whether they hunt and capture prey during the night is still unknown though. Interestingly, one species, *Copula sivickisi*, from the Indo Pacific is strictly night active and sits inactive and attached to the substrate during the day (Garm et al., 2012). This species is predominantly associated with coral reefs where it hunts a variety of planktonic crustaceans in the surface waters at night. Like *T. cystophora* they have internal fertilization and mating happens in the dark only (Hartwick, 1991; Lewis and Long, 2005; Garm et al., 2012, 2015). Despite the strictly nocturnal behavior, they still have the same set of small eyes (Figure 1) as the day active *T. cystophora*. How they locate each other or prey items in the dark is unknown and could in principle be governed by random swimming and accidental encounters. But this would be rather inefficient, unless prey, and mate densities are very high. In a recent paper we suggested an alternative method (Garm et al., 2012). At least at Okinawa, Japan, *C. sivickisi* are co-localized with the bioluminescent dinoflagellate *Pyrocystis noctiluca* which is constantly triggered to emit light by encounters with a variety of planktonic crustaceans (Garm et al., 2012). We hypothesized that the medusa of *C. sivickisi* are attracted by the flashes of blue light and thereby aggregate in areas with high prey densities.

Here we test the hypothesis that *C. sivickisi* locate areas of high prey densities using the bioluminescent signals from *P. noctiluca*. We focused on the lens eyes since they are the only image forming eyes and examined their optics and used electroretinograms (ERGs) to investigate their receptor physiology, including spectral sensitivity and temporal resolution. Further, we conducted behavioral experiments to test if they are attracted by the flashes emitted by *P. noctiluca*. All the results clearly support the hypothesis.

## Materials and methods

### Experimental organisms

Medusa of *C. sivickisi* were collected using light traps in the harbor at Akajima, Okinawa, Japan, and brought back to Akajima Marine Science Laboratory (AMSL) where they were kept in 40–80 l tanks with seawater at 29°C and 33 psu, and fed *Artemia* or copepods daily. The traps attracted medusae of both sexes and all sizes (1.5–8 mm in diameter). The electrophysiological experiments on the temporal resolution was carried out at AMSL using 10 adult animals of both sexes, but for spectral sensitivity measurements, 15 juvenile medusae were brought back to University of Copenhagen and raised to mature size during 4–5 weeks. They were raised in a 50 l tank at 29°C and 33 psu and fed SELCO enriched *Artemia* daily. Circulation in the tank was created by aeration and half the water was exchanged every other week. The behavioral experiments were conducted at AMSL using adult males and females (bell diameter 8–10 mm) within 3 days of capture. The dinoflagellate, *P. noctiluca*, was caught at nighttime in the harbor using a 100 μm plankton net. After manually sorting them from the rest of the plankton samples they were kept in a 60 l tank under natural light conditions at AMSL.

### Anatomical model

As a basis for our analyses of visual optics, a geometrically accurate model was made of the two lens eyes and their position in the rhopalium. The model was based on histological sections as well as fresh rhopalia photographed from the front, the side and from above. The shape of excised fresh lenses, together with histological sections, were used to determine the position and dimensions of all optically relevant structures. The model was based on five rhopalia from five fully-grown medusae.

### Focal-length measurements in the lens eyes

Fresh lenses from the lens eyes were excised from the eye by tearing the retinal cup with two needles, one on either side of the lens. Roughly 50% of the attempts delivered seemingly intact lenses. The isolated lenses were placed in seawater on a microscope slide and covered by a cover-slip, which was supported to form a 1 mm deep cavity. The lens was arranged such that the longest axis was perpendicular to the incoming light, as would be the case in an intact eye. The microscope condenser was removed and a pinhole of 0.5 mm was placed 10 cm below the preparation. Images were then taken through a high numeric aperture objective (x50), focusing first at the lens equator and then at every 10 μm to a distance well below the depth of best focus. Measurements were performed on five lenses from lower lens eyes and three lenses from upper lens eyes.

### Electrophysiology

For measurements of the dynamic range and spectral sensitivity, extracellular ERG recordings were obtained from seven lower lens eyes and seven upper lens eyes originating form 11 adult individuals of both sexes. A maximum of two rhopalia were used per animal and only one eye from each rhopalium. Rhopalia were dissected from the animals by cutting the rhopalial stalk and afterwards they were transfered to a petri dish in the electrophysiological setup containing seawater (29°C, salinity 33 psu). A custom made glass suction electrode was placed on the edge of either the upper or the lower lens eye and suction was applied until a slight migration of pigment into the electrode was observed. The diameter of the electrode tip was 1–3 μm resulting in an impedance of 2–5 MOhm. Recordings were amplified 1000 times and filtered (0.1 Hz high pass, 1000 Hz low pass, and 50 Hz notch filter) via a differential AC amplifier (1700, A-Msystems Inc., WA) and recorded using a custom made program for Labview (Labview 8.5, National Instruments, TX). The light stimulus was provided by an ultra-bright white LED (Luxeon III star, Philips, San Jose, CA) placed in a Linos microbench system (Linos, Goettingen, Germany). The microbench was equipped with a series of neutral density filters and interference color filters (half width = 12 nm, CVI laser, Bensheim, Germany). The stimulus was presented to the eye using a 1 mm light guide close to the pupil to create a close to even illumination of the entire visual field.

The experimental protocol started with 15 min of dark adaptation. Then an intensity series was presented covering four log units in steps of 0.3 or 0.7 log units starting at the low intensity end (1.1 × 10^1^W/sr/m^2^). This was followed by an equal quanta (6 × 10^18^ photons/s/sr/m^2^) spectral series covering 410–680 nm in 20 steps and the protocol ended with a 2nd intensity series to ensure that the sensitivity had not changed during the experiment. Each stimulus lasted 25 ms and the stimuli were presented with 1 1/2 min in between. Only data from eyes lasting a full protocol, where the 2nd intensity series differed <15% from the 1st, were used for the analysis. This similarity between the two V-log I curves also shows that the initial dark adaptation, stimulus duration, and inter stimulus times were long enough to avoid a change in adaptational state during the experiments. The data were analyzed manually in the program Igor Pro 6.12A (Wavematrics, Lake Oswego, Oregon). The spectral data were transformed by the V-log I curve to obtain the relative sensitivity (see Coates et al., 2006 for details on this procedure).

The temporal resolution of the lens eyes was examined using flicker fusion frequency (fff) experiments. Five upper lens eyes and five lower lens eyes were presented with a sinusoidal stimulus for 10 s covering the frequency spectrum 0.5–20 Hz in 0.5 Hz steps while recording the ERG as described above. Initially, the eyes were adapted for 10 min to the mid intensity of the stimulus, which was followed by the sinusoidal stimuli starting at the low frequency end with 2 min at mid intensity between. A full protocol thus lasted 90 min. The recordings were analyzed using a fast forward fourier transformation on what equals five stimulus cycles. The returned value at the principle frequency was normalized and used to create an fff curve.

### Behavioral experiments

Nine fully grown medusae were placed in a 20 l tank with seawater at 29°C and 33 psu and without circulation. The experiment was conducted within the natural activity period of the medusae at 10 p.m. and the medusae had last been fed the night before. The tank was kept in a fully darkened room and the animals were left for 30 min to adjust to the tank. A similar tank next to the medusa tank with the same conditions held ~300 *P. noctiluca* caught between 24 and 48 h prior to the experiments. The two tanks were separated by 0.5 cm to minimize possible transfer of vibrations. After the 30 min, aeration was started in the *P. noctiluca* tank and continued for 2 min. The bubbling had a frequency of 2–3 Hz and triggered the bioluminescence immediately. The behavioral response to the bioluminescence was recorded using a Sony handycam (Sony DCR-HC44) under infrared light (IR-65LED, Loligo Systems, Denmark; peak wavelength = 850 nm, intensity at surface = 27.5 W/m^2^/sr). The video was analyzed in a custom made program for Matlab 2013b (Mathwork, Inc., Natick, MA, USA) which tracked the position of the medusae from 2 min before the onset of the bioluminescence to 2 min after with a 2 s time resolution. In a further analysis of the video recording the tank was divided in four equally sized horizontal sectors (#1 closest to the bioluminescence, #4 the furthest away) and it was noted in which sector each of the medusae were positioned again with a time resolution of 2 s.

## Results

### Morphology

In *C. sivickisi* the rhopalia carry the six eyes, as typical for cubozoans (Figures 1A,B). The rhopalia hang in the rhopalial niche suspended on a flexible stalk. Along with the heavy crystal in the distal end, this results in the rhopalium always keeping the same vertical orientation with the upper lens eye pointing straight upwards (Figures 1C–F). The sections of the lens eyes show that they have the same structure with a thin cornea, a slightly elliptic lens, a thin vitreous space, upright ciliary photoreceptors also holding the brown screening pigment, and retinal associated neurons (Figure 1G). The lens cells appear dead and devoid of organelles in the center while the peripheral cells facing the retina have nuclei and other organelles. The photoreceptors of the two lens eyes are very similar and have outer segments of 40–70 μm depending on the area of the retina, with the central ones being the longest. The outer segments form dense layers of microvilli arising from a single cilium (Figures 1G,H). Interestingly, the outer segments in both lens eyes have large empty swellings along the central axis appearing like holes in the retina (Figure 1G).

### Optics

**Figure 2:**
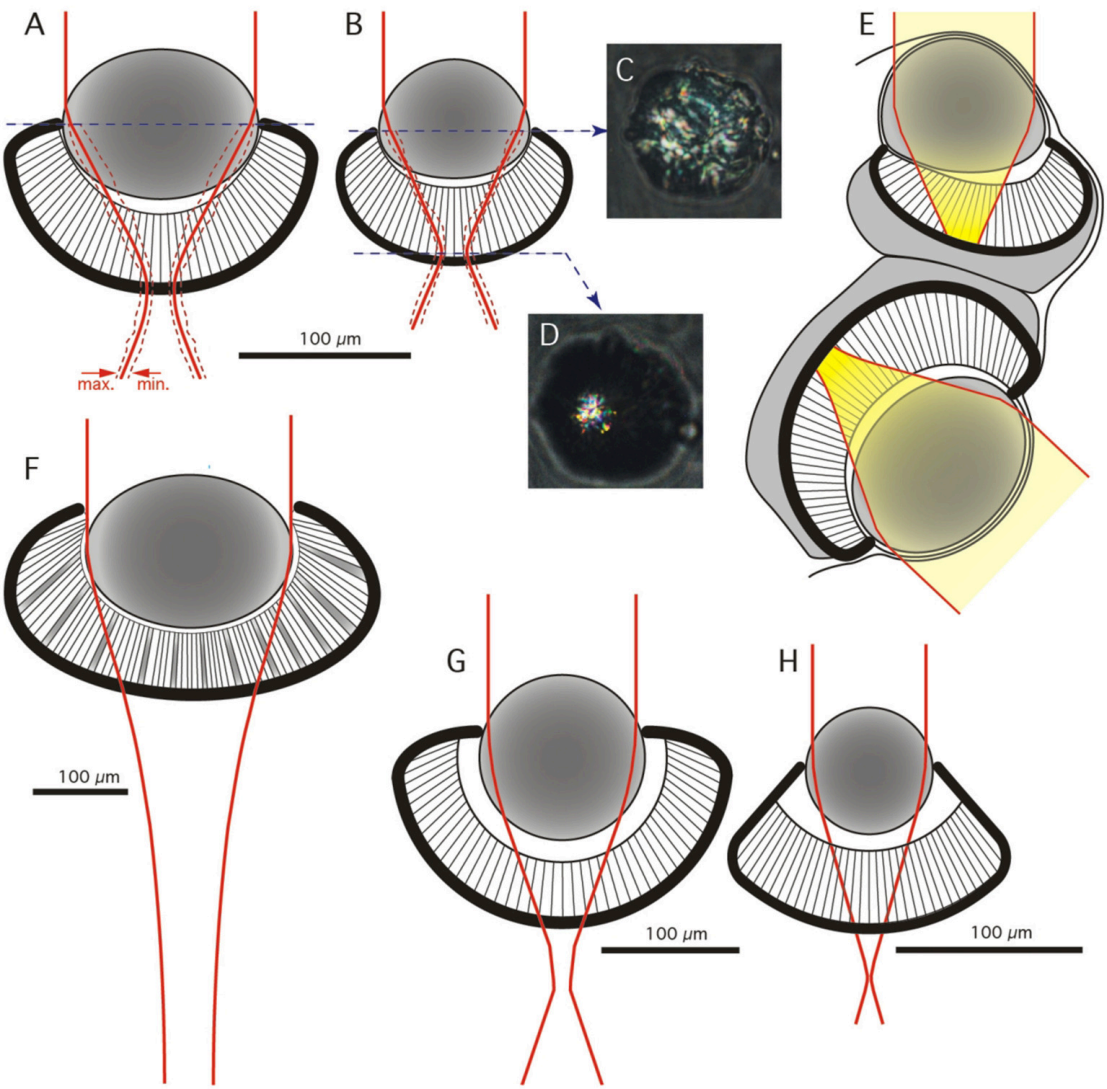
**Geometrical model and optics of the lens eyes. (A,B)** Frontal view of anatomical models of the lower **(A)** and upper (**B**) lens eye of *C. sivickisi*. Red line indicates average beam width of a distant point light source, broken red lines indicate maximum and minimum beam width (*N* = 5 in **A** and *N* = 3 in **B**). **(C,D)** Examples of the appearance of beam cross-sections behind a lens of the upper lens eye photographed at the lens equator and at the focal plane. **(E)** The average beam profilers from **(A,B)** plotted in the sagittal plane of the rhopalium. The yellow field highlights beam width for a point source and thus the blur spot in the retina. **(F)** Optical model of the lower lens eye of *Chiropsella bronzie* for comparison (modified from O'Connor et al., 2009). **(G,H)** Optical models of the lower and upper lens eyes of *Tripedalia cystophora* respectively (modified from Nilsson et al., 2005).

Isolated fresh lenses were slightly ellipsoidic with the longer axes in the pupil plane (Figures 2A,B). Using a compound microscope to project parallel light through the lens we determined the focusing properties of the lens by measuring the width of the beam as a function of distance behind the lens. Lenses from both the upper and lower eyes brought light to a focus at a surprisingly short distance—approximately 100 μm. At the plane of best focus, the beam was converged to a diameter of 15–20% of the lens diameter. To account for the variation in eye size and lens size (about ± 25%) we normalized all measurements to units of lens diameter and plotted the beam profile in an anatomical model of the eye (Figures 2A–E). This demonstrated that the plane of best focus is at the base of the retina in both the upper and lower lens eyes. Even though the f-number (focal ratio) is lower than that found in the related jellyfish *T. cystophora*, we estimate that the spatial resolution will be roughly the same, i.e., 10–20° in both upper and lower lens eyes (compare Figures 2A,B,F–H).

### Dynamic range

**Figure 3:**
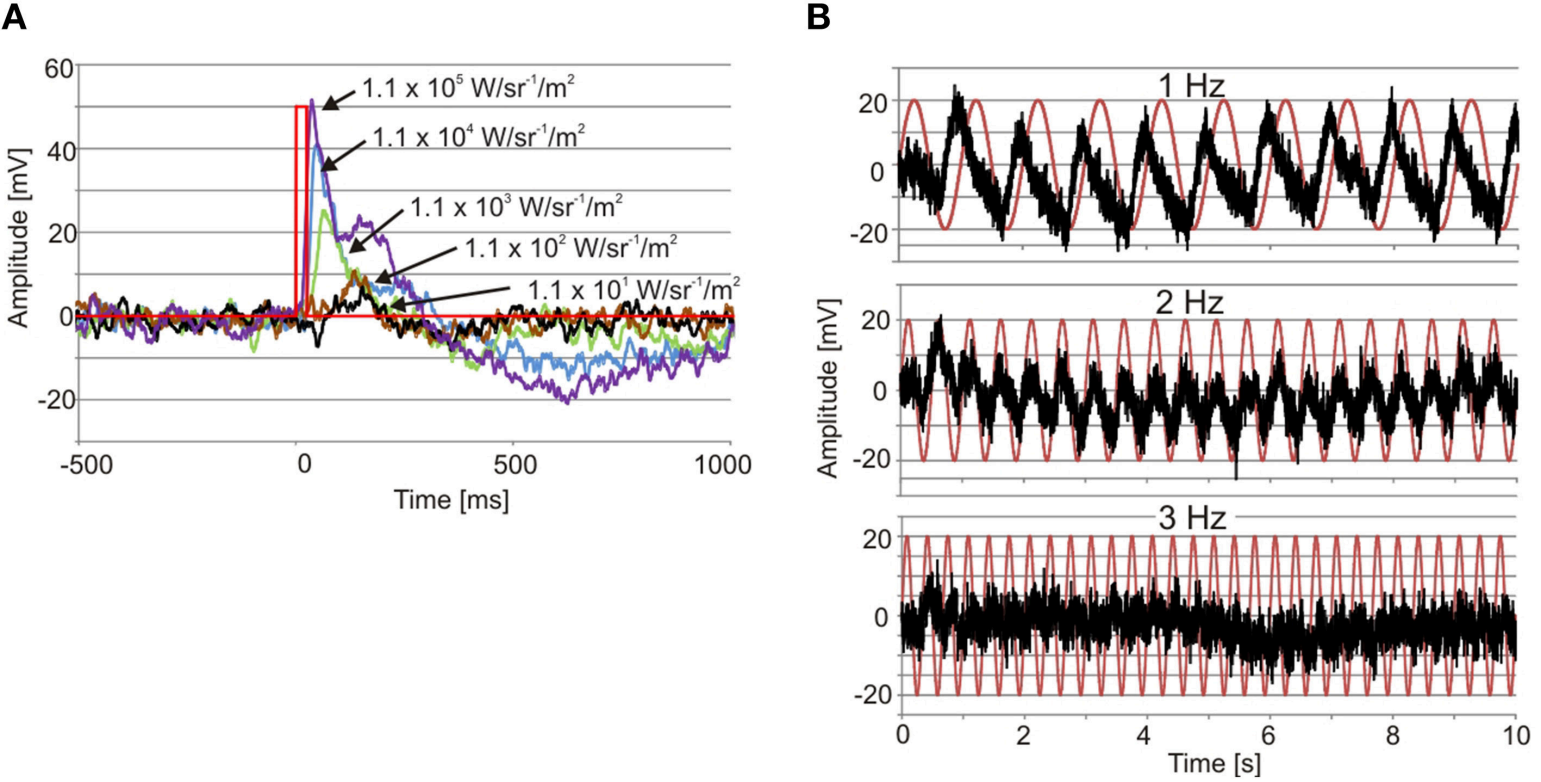
**Examples of ERGs. (A)** Recordings from an upper lens eye showing the graded impulse response to 25 ms flashes of light (red line) covering four log units. Note also the longer time to peak with declining light intensity. **(B)** Recording from a lower lens eye showing the responses to 1, 2, and 3 Hz sinusoidal light stimuli (red traces). Note that the response peaks before max intensity at 1 Hz.

**Figure 4:**
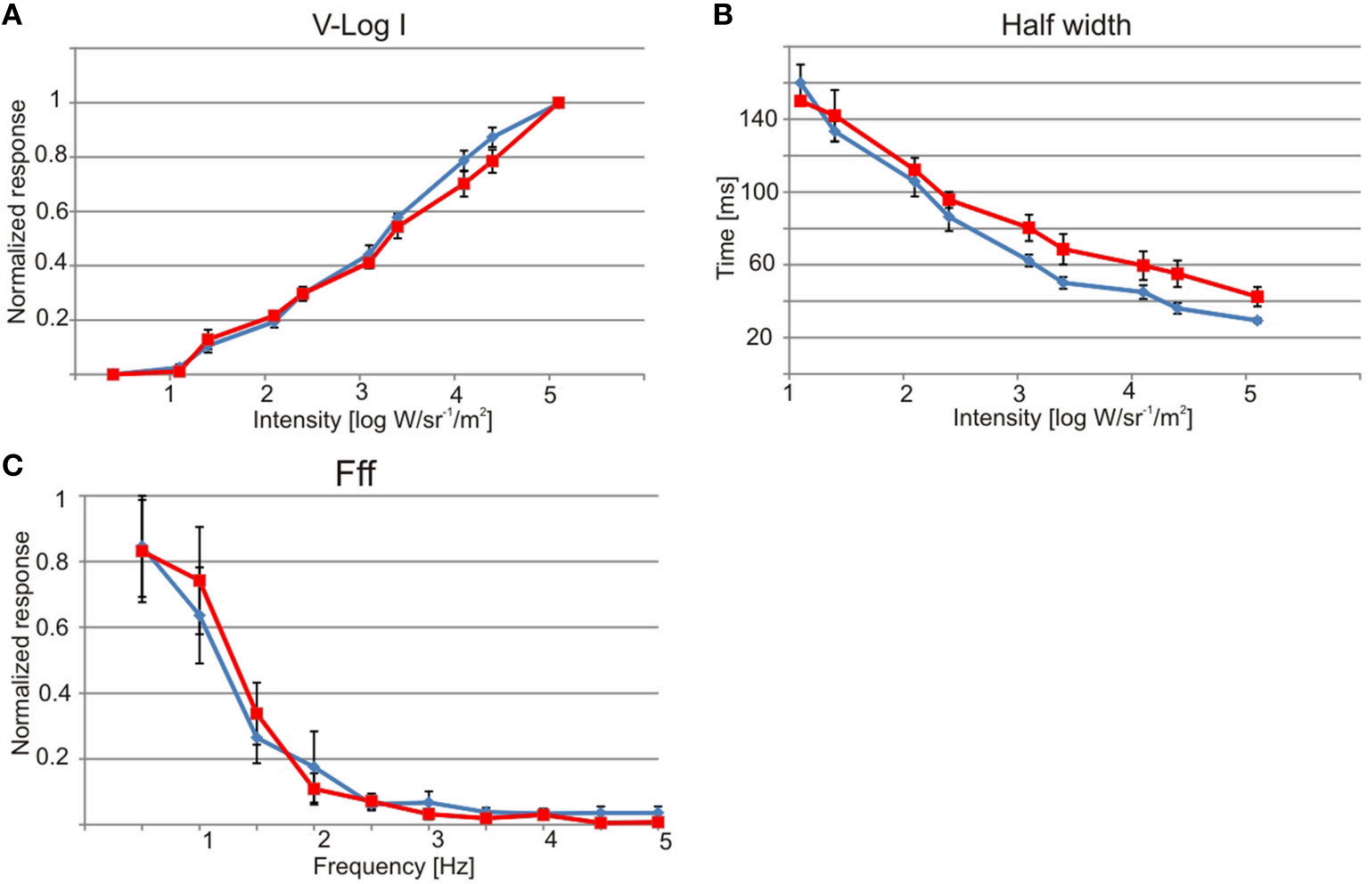
**Physiology of the lens eyes**. Upper lens eye (ULE) in blue and lower lens eye (LLE) in red. **(A)** V-log I curve showing a dynamic range of about four log units from ~10 to 1 × 10^5^ W/sr/m^2^. **(B)** The half width of the impulse response decrease with increasing intensity. At max intensity the half width was 29 ms for ULE and 42 ms for LLE. **(C)** Both lens eyes are very slow and had flicker fusion frequencies (fff) of about 2.5 Hz. All curves are showing mean ± S.E.M. *N* = 7, except for **(C)** where *N* = 5.

The electrophysiologically recorded dynamic range was very similar in the upper and lower lens eyes, and flashes of light with varying intensity resulted in graded impulse responses typically biphasic (Figure 3A). The dynamic range covered at least four log units from 1.1 × 10 to 1.1 × 10^5^ W/m^2^/sr (Figures 3A, 4A). It might well be broader tough, since the V-Log I curves showed no sign of saturation in the high intensity end (Figure 4A).

### Spectral sensitivity

**Figure 5:**
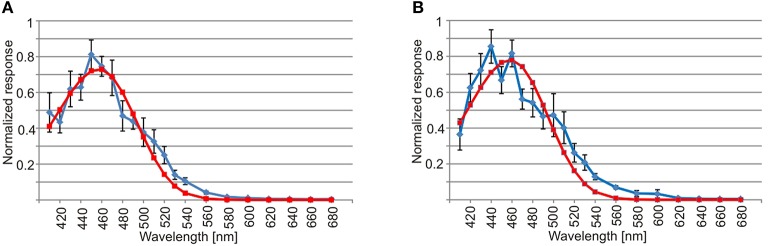
**Spectral sensitivity of the lens eye. (A)** The spectral sensitivity curve of the upper lens eye (blue line) has a single peak in the deep blue part of the spectrum and has a good match with the absorption curve of a 458 nm opsin (red line, AIC = −53.7). **(B)** Similar to the upper lens eye the spectral sensitivity of the lower lens eye (blue line) peaks in the deep blue part of the spectrum and had a best match with a single opsin peaking a 458 nm (red line, AIC = −35.9). The curves are showing mean ± S.E.M., *N* = 7.

The spectral sensitivity curves were also very similar in the two lens eyes. They had a single peak in the deep blue part of the visual spectrum around 460 nm (Figure 5). Using the least square of the mean method to fit the spectral sensitivity curve of the lens eyes to theoretical absorption curves of opsins (Govardovskii et al., 2000), returned the best match in both lens eyes to a single opsin peaking at 458 nm (Figure 5). In contrast to linear models where the *R*^2^ is commonly used, the goodness of fit for non-linear models is best described by Akaike's Information Criterion (AIC). The AIC for the opsin fit was −53.7 and −35.9 for the upper and lower lens eye, respectively. Note that more negative values reflect a better model fit.

### Temporal resolution

The temporal resolution of the two lens eyes was tested using two different methods, a direct and an indirect. In the indirect method the width at half height of the impulse response was measured, indicating a difference between the two eyes. The upper lens eye showed a decreasing half width with increasing light intensity of the 25 ms flashes, and at the highest intensity the half width was 29 ± 0.5 ms (mean ± SEM; Figure 4B). The lower lens eye was slower at all tested intensities and had a minimal half width of 42 ± 4 ms (mean ± SEM; Figure 4B). The flicker fusion frequency (fff) experiments provided stable responses to the sinusoidal stimulus throughout the entire stimulation period (Figure 3B). Interestingly, when measuring the temporal resolution with this direct method the difference between the upper and lower lens eyes disappeared. Both eyes were very slow and showed a close to linear decline in the response to a sinusoidal flicker going from 0.5 to 2.5 Hz (Figures 3B, 4C). Above 2.5 Hz no response was seen and the fff is thus ~2.5 Hz for both eyes. At low frequencies, <1.5 Hz the response peak preceded the stimulus peak putatively due to build in temporal filters. The same is seen for *T. cystophora* (O'Connor et al., 2010).

### Response to bioluminescence from *Pyrocystis noctiluca*

**Figure 6:**
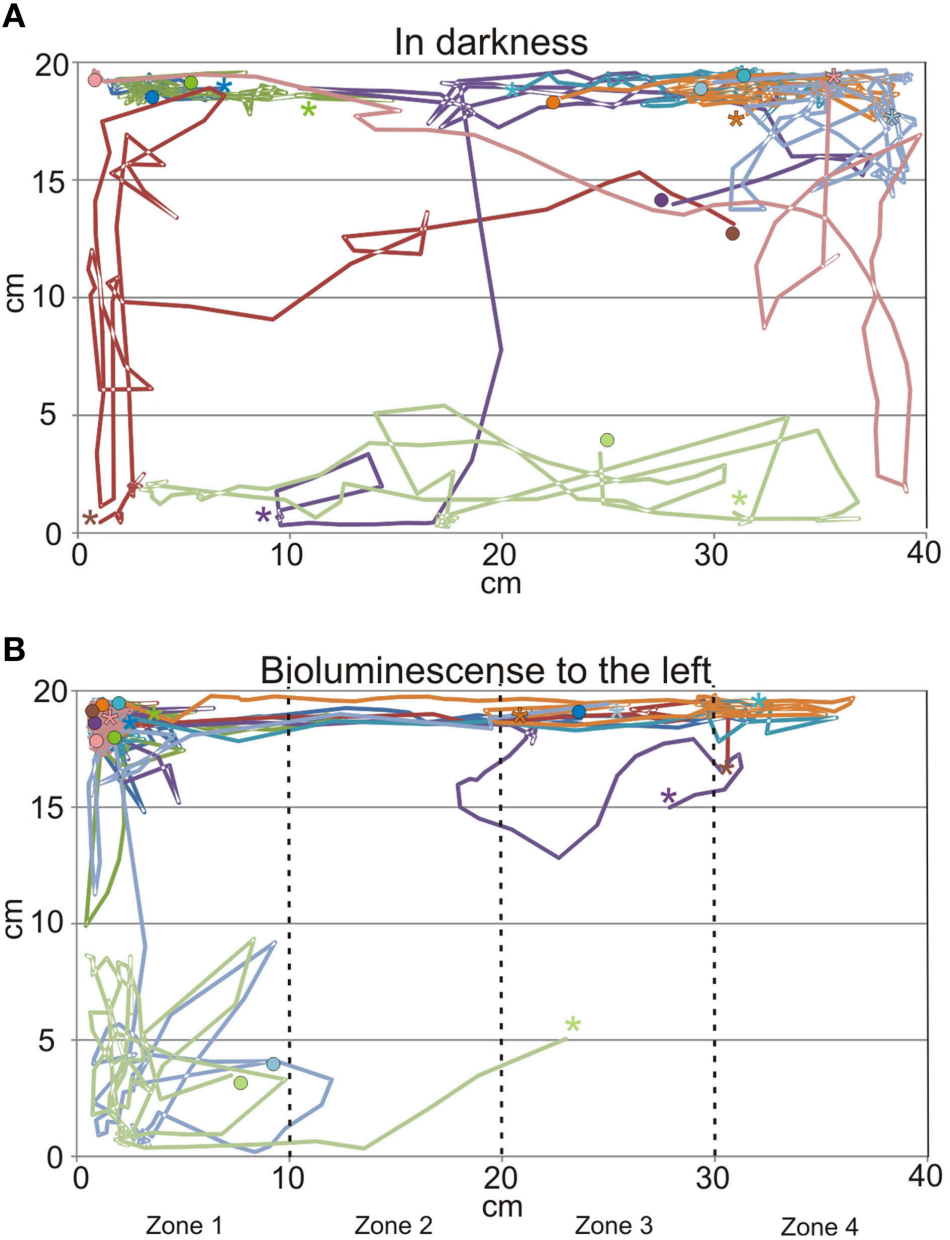
**Swim trajectories from behavioral experiments. (A)** In darkness the medusae swim in most of the tank but had a preference for the corners of the tank. **(B)** When the dinoflagellate *Pyrocystis noctiluca* is emitting bioluminescent light to the left of the tank the medusae spend most of the time in the left side of the tank. Each trace represents 2 min with 2 s time resolution and each color represents a single medusa (*N* = 9). It is the same nine medusae in **(A,B)** and timewise the trajectories from **(A)** continues in **(B)**. See also Figure 7. Note the parallel run of the blue and green trance in the bottom left where a mating started. ^*^ indicates start of trajectory, • end.

**Figure 7:**
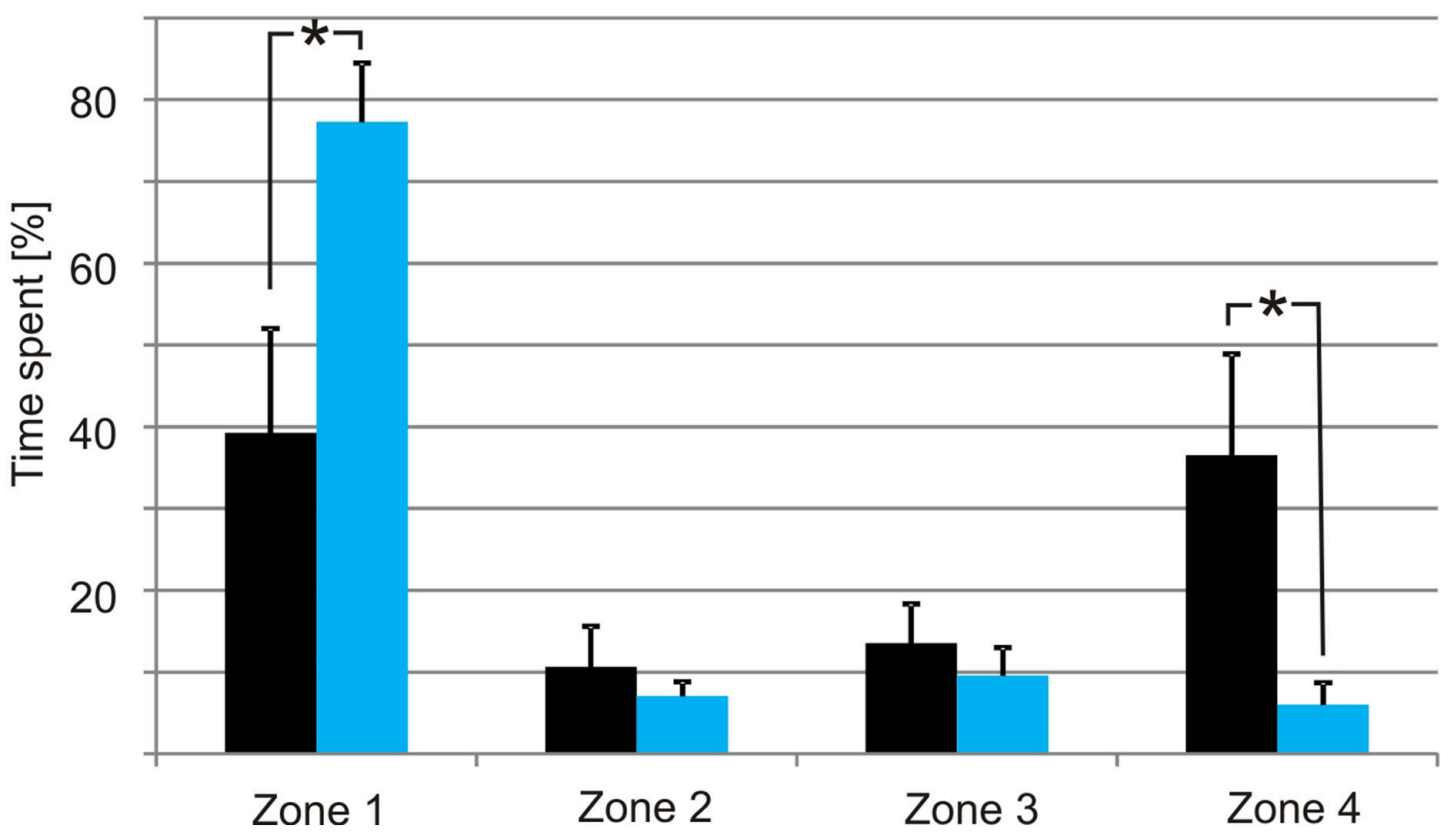
**Distribution of the medusae in darkness (black bars) and with bioluminescence (light blue bars)**. The tank was divided into four zones with zone 1 closest to the tank with *P. noctiluca* and zone 4 the furthest away. In darkness the medusae spend most time at the ends of the tank but with no significant difference between zone 1 and zone 4. When the bioluminescence of the algae is activated the medusae spend most of the time in zone 1. Asterisks indicate significant difference at the 0.05 level. Bars are means ± S.E.M., *N* = 9.

When the tank holding the nine medusae was kept in darkness, the medusae displayed what we consider to be a natural foraging behavior, swimming slowly with their tentacles fully or partly extended near the surface. They did not distribute evenly but preferred the ends of the tank over the middle part (Figures 6A, 7), which is a natural consequence of random exploratory behavior. Importantly, they spent the same amount of time in the two ends (two-sided unpaired student *t*-test, *p* = 0.88). There was a marked change in behavior soon after the aeration was turned on and the bioluminescence initiated (Figure 6B). Some medusae swam directly to the end of the tank toward the bioluminescence and stayed there throughout the 2 min. Others took 10–20 s before swimming toward the light but all medusae spend most of the 2 min in zone 1 close closest to the bioluminescence flashes (Figure 6B). In the 2 min with bioluminescence the medusae spent significantly more time in zone 1 than any of the other zones of the tank (two-sided unpaired student *t*-test, *p* > 0.0001). They also spent significantly more time in zone 1 during activation of the bioluminescence than in darkness and less time in zone 4 (two-sided unpaired student *t*-test, *p* = 0.027 and 0.028, respectively). At the end of the 2 min, two medusae started mating (Figure 6B, parallel blue and green trace).

## Discussion

Our results show that the lenses of both lens eyes of *C. sivickisi* form under-focused images on the retinae, generating poor spatial resolution with large blur spots in the range of 10–20° depending on retinal location. Further, the eyes are color-blind, having a single opsin with peak sensitivity in the blue part of the spectrum close to 460 nm. Both lens eyes have very low temporal resolution with fff's around 2.5 Hz. We also show that under dark conditions the medusae are attracted to bioluminescent flashes produced by the dinoflagellate *P. noctiluca*, which is occurring in high densities in their natural habitat. In conclusion all data support our hypothesis, that the medusae use vision to find parts of the habitat where hunting will be most successful. They are unlikely, however, to use vision to directly spot or pursue individual prey items due to their low spatial resolution.

### Low intensity vision for spotting bioluminescence

So far *C. sivickisi* is the only cubomedusa known to be strictly night active (Garm et al., 2012). Still, they have a visual system similar to all other examined cubomedusae. Our morphological data show that the lens eyes of *C. sivickisi* are structurally similar to other box jellyfish eyes (Claus, 1878; Berger, 1900; Yamasu and Yoshida, 1976; Pearse and Pearse, 1978; Nilsson et al., 2005). Fully grown, the upper and lower lens eyes have a diameter of about 150 and 200 μm, respectively. In general, small eyes with pupils in the order of 100 μm provide only low spatial resolution, especially at the low light intensities present at night. But our results also point to several features, both in the morphology and in the physiology, which will enhance the photon capture. The outer segments are relatively long and have very dense membrane stacking when compared to other box jellyfish (Laska and Hündgen, 1982; Martin, 2004; O'Connor et al., 2010). Assuming that this implies more photopigment, it will enhance the photon capture, and can thus be interpreted as adaptation for a nocturnal life-style. If there is more opsin per photoreceptor cell it will also result in additional dark noise, which will set a limit to the dimmest stimulus that can be discriminated from noise (Barlow, 1956).

The photon capture is further optimized by a long integration time shown by the very low temporal resolution with flicker fusion frequencies of about 2.5 Hz. Such long integration time is typically found in night active or deep sea animals and are adaptations for vision in low light intensities (Warrant, 2004; Warrant and Locket, 2004). The long integration times has the obvious disadvantage of causing motion blur of objects moving across the visual field. The large acceptance angles of the photoreceptors, estimated to be 10–20°, will also enhance photon capture but reduce the ability to spot individual bioluminescent flashes. This supports the notion that the lens eye of *C. sivickisi* are tuned for finding the direction toward the densest population of bioluminescent organisms rather than guiding behavior toward single bioluminescent flashes. Detection of bioluminescence is further supported by the spectral sensitivity of the lens eyes peaking close to 460 nm which is a fairly good match with the peak emission of 473–478 nm from *P. noctiluca* (Hastings and Morin, 1991). Further, the flashes typically have a duration between 100 and 200 ms which is slightly faster than the temporal resolution we find for both lens eye and this ensures a maximum photon capture from each flash (Hastings and Morin, 1991).

The slight mismatch between the spectral sensitivity of the photoreceptors and the *P. noctiluca* emission is probably hinting at another important visual task for the medusae. At dawn the medusae come to a rest and anchor themselves to the underside of stony corals (Garm et al., 2012). This means that they have to seek out the coral in the morning light and having a spectral sensitivity in the deep blue part of the spectrum will optimize the contrast between the reef structures and the surrounding ocean water. Clear ocean water peaks at about 450 nm (McFarland and Munz, 1975) whereas coral reef structures, though varying, typically reflect very little blue light and much more in the green and red part of the spectrum (Schalles et al., 2000; Hochberg et al., 2004). The peak sensitivity at about 460 nm could thus be seen as a compromise allowing both habitat recognition and prey detection.

### Retinal cavities for noise reduction

As mentioned above, dark noise is a major problem for vision at low light intensities. Interestingly, the retinal structure with a large fraction (about 50%) of empty spaces found in *C. sivickisi* might be a unique way to minimize this problem. In the closely related diurnal species *T. cystophora* the lens eyes have very similar shapes and sizes, but there is much less empty space in the retina (<10–15%; Nilsson et al., 2005). There is a possibility that the retinal cavities are part of the adaptation to a nocturnal life-style. The longitudinal structure of the empty spaces will concentrate light to the microvillar sections between the spaces, because these have a higher refractive index and will trap some light by total internal reflections. This will boost absorption in the photopigment. The spaces will also reduce the microvillar volume of the retina, and if this means fewer rhodopsin molecules it will lead to less thermal noise. We thus hypothesize that the pronounced cavities in the *C. sivickisi* retina is yet another adaptation for a nocturnal life style, providing both a better signal and less noise. However, without modeling the ray path in the retina and measuring both density and thermal instability of the rhodopsin, it is not possible to assess to which degree the retinal spaces will improve the signal to noise ratio.

### Visually guided hunting in *C. sivickisi*

Like many other cnidarian medusae, the medusa of *C. sivickisi* is a predator feeding for a large part on pelagic crustaceans (Larson, 1976; Mackie, 1980; Buskey, 2003; Colin et al., 2003; Garm et al., 2012). While most medusae behave as plankton organisms following the currents, cubomedusae are agile swimmers actively choosing their location. Even though a higher number of test animals than used here would be needed to understand the full details of the hunting behavior of *C. sivickisi*, the statistical significance of our results show that they use vision to place themselves in areas with maximum prey density. This they do using the bioluminescence emitted when their crustacean prey contact the dinoflagellate *P. noctiluca* which can be present in high densities in the habitat. The observed mating during the experiments indicates that the results could be influenced by a group effect (tendency to aggregate). Since the medusae in general displayed natural behavior (including mating) in the tank and since they are also found in close vicinity to conspecifics when hunting in the natural habitat we trust that even though a group effect might have been present it has not resulted in unnatural behavior during the experiments.

The hunting behavior of *C. sivickisi* is similar to the diurnal box jellyfish *T. cystophora*, which is visually attracted to light shafts produced by gaps in the mangrove canopy. These light shafts also attract their crustacean prey, and when these occur in large numbers, their light scattering effect makes the light shafts much more visible. The role of vision in *C. sivickisi* and *T. cystophora* is not to spot individual prey animals, which is typically implied by the term “visually guided predation.” But both species are clearly engaged in visually guided foraging, and it may be the passive mode of prey capture in medusae that have prevented the evolution of high resolution vision in box jellyfish. In principle terms, the foraging mode of *C. sivickisi* and *T. cystophora* is an important example of a visual task that may drive evolution from low resolution to high resolution vision. It can be seen, therefore, as an important intermediate between simple low resolution vision for habitat selection and the high resolution vision which putatively drove the evolution of large eyes and brains in vertebrates, cephalopods and arthropods (Nilsson, 2009, 2013).

## Author contributions

AG designed the experiments, conducted some of the experiments, wrote the initial draft of the MS incl the figures, and financed most of the work. JB conducted some of the experiments. RP conducted some of the experiments. DN conducted some of the experiments and financed part of the work. All authors helped finalize the MS.

### Conflict of interest statement

The authors declare that the research was conducted in the absence of any commercial or financial relationships that could be construed as a potential conflict of interest.
